# Ten-year trends of utilizing palliative care and palliative procedures in patients with gastric Cancer in the United States from 2009 to 2018 - a nationwide database study

**DOI:** 10.1186/s12913-021-07404-1

**Published:** 2022-01-04

**Authors:** Moon Kyung Joo, Ji Won Yoo, Zahra Mojtahedi, Pearl Kim, Jinwook Hwang, Ja Seol Koo, Hee-Taik Kang, Jay J. Shen

**Affiliations:** 1 School of Public Health, University of Nevada Las Vegas, Las Vegas, NV USA; 2 Division of Gastroenterology, Department of Internal Medicine, Korea University Guro Hospital, Korea University College of Medicine, Seoul, 08308 Republic of Korea; 3 Department of Internal Medicine, School of Medicine, University of Nevada Las Vegas, Las Vegas, NV USA; 4 Department of Healthcare Administration and Policy, School of Public Health, University of Nevada, Las Vegas, NV 89119 USA; 5 Department of Thoracic and Cardiovascular Surgery, Korea University Ansan Hospita, Korea University College of Medicine, Ansan, Republic of Korea; 6 Division of Gastroenterology, Department of Internal Medicine, Korea University Ansan Hospita, Korea University College of Medicine, Ansan, Republic of Korea; 7 Department of Family Medicine, Chungbuk National University Hospital, Chungbuk National University College of Medicine, Cheongju, Republic of Korea

**Keywords:** Palliative care, Gastric cancer, Hospital costs, Hospice, Palliative procedure, Length of stay

## Abstract

**Objectives:**

Little is known about the current status and the changing trends of hospitalization and palliative care consultation of patients with gastric cancer in the United States. The aim of this study was to evaluate the changing trend in the number of hospitalization, palliative care consultation, and palliative procedures in the US during a recent 10-year period using a nationwide database.

**Methods:**

This was a retrospective study that analyzed the National Inpatient Sample (NIS) database of 2009–2018. Patients aged more than 18 years who were diagnosed with a gastric cancer using International Classification of Diseases (ICD)-9 and 10 codes were included. Palliative care consultation included palliative care (ICD-9, V66.7; ICD-10, Z51.5) and advanced care planning (ICD-9, V69.89; ICD-10, Z71.89). Palliative procedures included percutaneous or endoscopic bypass, gastrostomy or enterostomy, dilation, drainage, nutrition, and irrigation for palliative purpose.

**Results and discussion:**

A total of 86,430 patients were selected and analyzed in this study. Using a compound annual growth rate (CAGR) approach, the annual number of hospitalizations of gastric cancer patients was found to be decreased during 2009–2018 (CAGR: -0.8%, *P* = 0.0084), while utilization rates of palliative care and palliative procedures increased (CAGR: 9.3 and 1.6%, respectively; *P* < 0.0001). Multivariable regression analysis revealed that palliative care consultation was associated with reduced total hospital charges (−$34,188, *P* < 0.0001).

**Conclusion:**

Utilization of palliative care consultation to patients with gastric cancer may reduce use of medical resources and hospital costs.

**Supplementary Information:**

The online version contains supplementary material available at 10.1186/s12913-021-07404-1.

## Introduction

Gastric cancer is the 3rd leading cancer-related cause of death with more than 720,000 deaths per year worldwide. The global incidence of gastric cancer is the highest in East Asian countries such as South Korea and Japan [1]. As socioeconomic levels and hygiene status are improved with universalized eradication therapy of *Helicobacter pylori*, the strongest etiologic factor of stomach cancer, the global incidence of gastric cancer is steadily declining [2]. With dramatic improvement of endoscopic techniques for visualization and resection of early gastric cancer (EGC), early detection and curative endoscopic resection of EGC have made a significant contribution to the decline of cancer-related mortality [3]. However, if gastric cancer is diagnosed at a later stage with distant metastasis, chance for cure declines dramatically and overall prognosis remains poor. A previous study has shown that if gastric cancer is diagnosed at stage III or IV, overall 5-year survival rate is only 35% regardless of surgical resection of tumor [4].

Gastric cancer is not considered as a major cancer in terms of incidence and mortality in the United States. However, recent data have shown that the incidence of gastric cancer in the US is 6.6 per 100,000 population with a morality rate of 3.3 per 100,000 during 2012–2016, imposing a burden to the healthcare system [5]. Unfortunately, very limited studies have evaluated demographic descriptive data and changing trends of inpatient hospitalization of gastric cancer patients [6]. Furthermore, little is known about the current status and changing trend of palliative care for gastric cancer patients, particularly patients who are near end of life (EOL). Palliative care is a comprehensive approach to manage serious illness such as terminal malignancy and other chronic ill diseases. It is known to be effective for improving the quality of life and avoiding unnecessary invasive procedures [7]. With this background, the objective of this study was to investigate changing trends of hospitalization, palliative care consultation, and palliative procedures for gastric cancer patients in the US from 2009 to 2018 using a nationwide database. We will also examine factors associated with utilization of palliative care consultation and palliative procedures and factors influencing hospital charges of gastric cancer patients during hospitalization.

## Methods

### Study design and data source

A serial, cross-sectional retrospective analysis was conducted using discharge data from the National Inpatient Sample (NIS) dataset. NIS database is developed as a part of the Healthcare charge and Utilization Project (HCUP). It is sponsored by the Agency for Healthcare Research and Quality (AHRQ). A comprehensive overview of NIS database is available at https://www.hcup-us.ahrq.gov. NIS includes approximately 20% stratified sample of discharges of community hospitals from over 40 states in the US. It currently contains data from more than seven million hospital stays each year. Inpatient data contained in the NIS represent more than 97% of inpatient hospitals from community hospitals in the US [8]. NIS also contains random samples of hospitalizations classified by HCUP member hospitals and stratified by location, teaching status, and bed size as indicated on the American Hospital Association Annual Survey of Hospital [9]. Upon completion of a data user agreement with the AHRQ, completely de-identified data were used for analysis of NIS. It was practically not feasible to receive patient consent from a deidentified large secondary dataset. The federal government intentionally made it impossible to link any hospital discharge in NIS with the state the patient resides in to protect the patient’s privacy and confidentiality. The Institutional Review Board (IRB) at the University of Nevada Las Vegas (UNLV) found that the data included in the analysis is deidentified thereby safeguarding privacy and confidentiality concerns and the current study to be exempt (IRB no. 1098939–3).

### Patient cohort selection and variables

**Fig. 1:**
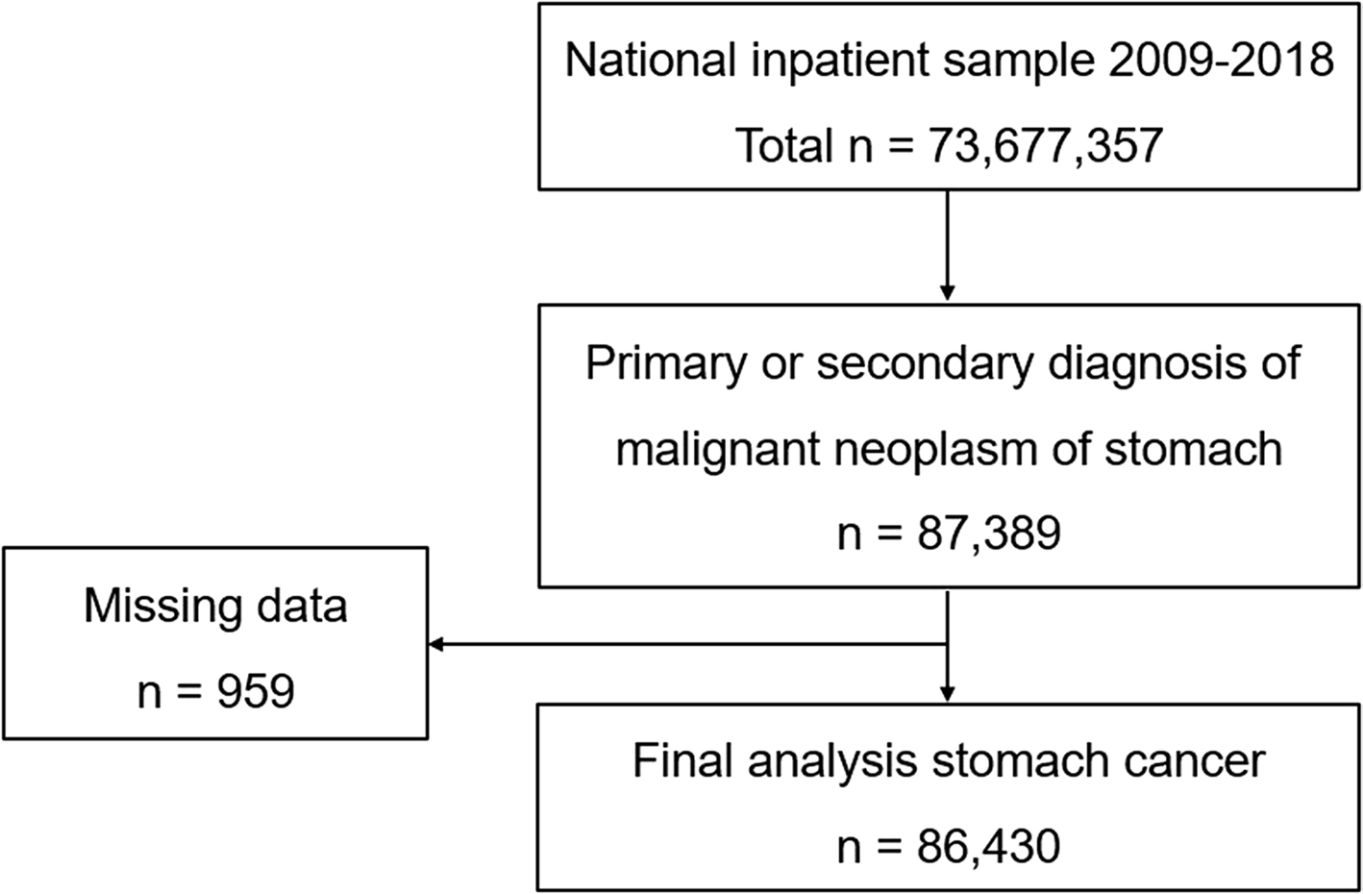
Algorithm for Selecting the Patient Cohort

NIS datasets from 2009 to 2018 were analyzed. Our population of interests were adult patients aged ≥18 years who had a primary or secondary diagnosis of malignant neoplasm of stomach from 2009 to 2018 in the US. International Classification of Diseases, 9th revision. Clinical Modification (ICD-9-CM) codes (151.x), and ICD-10-CM codes (C16.x) were used to identify gastric cancer patients. The selection process of patient cohort is summarized in Fig. 1. Among 73,677,357 of NIS data sets from 2009 to 2018, 87,389 patients with gastric cancer as a primary or secondary diagnosis were identified. After excluding 959 patients with missing data, a total of 86,430 patients were included for the final analysis with the national estimate of weighted number of 441,004. NIS data for years prior to 2012 includes both hospital and discharge weights. The hospital weights can be used to produce hospital-level estimates, and the discharge weights can be used to produce discharge-level estimates. NIS data for years 2012 onwards, should only be weighted to produce discharge-level estimates. Therefore, the change in 2012 did not affect our national estimate that was based on the discharge weight only.

Palliative procedures included percutaneous or endoscopic procedures of bypass, gastrostomy or enterostomy, dilation, drainage, nutrition, and irrigation for palliative purpose. They were identifying using ICD-9-CM and ICD-10-PCS codes (Supplementary Table 1). Palliative care consultation included palliative care (ICD-9, V66.7; ICD-10, Z51.5) and advanced care planning (ICD-9, V69.89; ICD-10, Z71.89). They were validated as optimal methods for identification of palliative care services in previous studies [10, 11]. Sociodemographic characteristics for each patient included gender, age, race, payer source (Medicare, Medicaid, private insurance, uninsured, no charge and other), quartile of median household income by zip codes, the severity of illness (All Patient Refined Diagnosis-Related Group [APR-DRG]), palliative care consultation, the number of diagnoses, palliative procedures, length of stay (LOS), total charges, and in-hospital death. It should be noted that since individual household income is not available in the NIS, AHRQ includes the quartile of median household income by zip codes in the NIS to reflect socioeconomic environments where patients reside. This variable has been widely used in many NIS-based clinical outcome research studies that have covered a variety of clinical conditions [12–14] Hospital characteristics included hospital size, hospital locations, teaching hospitals, and regions. The primary outcomes of our analysis were proportion and trends of palliative care and palliative procedures, and the secondary outcomes were factors associated with utilization of palliative care and palliative procedures, and how they affected total hospital charges among gastric cancer patients. Total hospital charges were calculated after adjusting for annual increase rate of hospital care expenditures published by the Centers for Medicare and Medicaid Service [15, 16].

### Statistical analyses

Compound annual growth rate (CAGR) was used to quantify temporal trends of annual number of hospitalizations, palliative care, and palliative procedures. CAGR was calculated as (y/x)^[1/(B-A)]-1^, where year A was x and year B was y [9, 17]. Statistical analysis was performed using Rao-Scott correction for χ^2^ tests for categorical variables. Patient and hospital characteristics are presented as mean (standard deviation) or percentages.

Generalized multiple logistic regression models taking patient and hospital characteristics into account were used to examine trends of palliative care consultation and palliative care procedures as well as potentially associated clinical factors. Odds ratios (ORs) and their corresponding 95% confidence intervals (CIs) were calculated for predictors. How palliative care and palliative procedures affected total hospital charges was also investigated using the generalized multiple linear regression analysis considering the potential of within hospital variations. All analyses were performed using SAS statistical software version 9.4 (SAS Institute Inc., Cary, NC, USA). All reported *P*-values are two sided. A *P*-value < 0.05 was considered statistically significant.

## Results

### Characteristics of patient cohort

Table 1 shows patient and hospital characteristics of NIS from 2009 to 2018. Among 86,430 of enrolled patients, males (63.4%) were predominant. Their mean age was 66.7 years with a standard deviation of 14.1 years. More than half (56.8%) of patients were white with Medicare. Most patients were admitted to large hospitals (62.9%) located in rural areas (67.2%). Almost two thirds of the cohort belonged to APR-DRDG 3 and 4 categories (46.3 and 17.8%, respectively). A total of 12.1% of patients received palliative care consultation. Palliative procedures were performed in 13.5% of patients. Total hospital charge was $92,052 ± 13,585. Mean LOS was 7.7 ± 8.4 days. It was found that 7.2% of patients died in hospitals. When cross-sectional data of years 2009, 2012, 2015, and 2018 were compared, palliative care consultation (7.1, 10.6, 14.0, and 17.4%, respectively) and number of diagnoses (10.8, 12.6, 14.5, 16.2) showed an increasing trend over time, while LOS (8.2, 7.9, 7.6, 7.1 days) and total charges ($95,921, $95,555, $92,912, $86,085) showed a decreasing trend.

**Table 1:** Patient and Hospitalization Characteristics in Gastric Cancer (2009–2018 NIS)

Year	2009–2018	2009	2012	2015	2018
n	86,430	8606	8648	8447	8088
Weighted N (national estimate)	441,004	45,034	44,285	43,055	41,115
Sociodemographics					
Gender					
Male	63.4	62.9	62.9	64.1	63.8
Female	36.6	37.1	37.1	35.9	36.2
Age, mean years (SD)	66.7 (14.1)	66.1 (14.1)	65.8 (14.1)	65.6 (13.9)	65.6 (13.9)
Age group					
< 30	1.0	0.6	1.0	0.9	0.9
30–39	3.4	3.2	3.2	3.5	3.8
40–49	8.5	8.9	8.3	8.1	8.5
50–59	18.9	18.1	19.7	19.3	17.7
60–69	26.1	25.0	25.9	26.9	27.5
70–79	24.3	24.9	23.3	23.8	24.8
≥80	17.7	19.2	18.7	17.4	16.8
Race					
White	56.8	59.4	58.9	56.3	52.2
Black	16.5	15.3	16.3	17.6	16.9
Hispanic	15.2	13.3	13.6	14.9	17.8
Asian/Pacific Islander	7.0	7.5	6.4	7.3	7.4
Native Americans/others	4.6	0.6	0.5	0.5	0.7
Payer source					
Medicare	52.5	51.5	53.4	52.8	52.7
Medicaid	12.9	10.1	12.1	13.2	15.0
Private insurance	28.5	32.3	28.0	28.5	26.8
Uninsured	3.0	3.6	3.5	2.4	2.8
No charge	0.4	0.4	0.3	0.5	0.3
Other	2.6	2.2	2.8	2.7	2.4
Median household incomes by zip code					
76th to 100th percentile	28.7	27.2	28.8	30.1	28.6
51th to 75th percentile	24.3	25.2	23.0	22.7	25.9
26th to 50th percentile	23.7	23.2	23.5	23.9	23.5
0th to 25th percentile	23.3	24.4	24.7	23.4	22.0
Hospitalization					
Severity of illness					
APR-DRG 1	3.9	4.7	4.6	3.1	3.2
APR-DRG 2	32.0	35.6	35.7	29.7	25.6
APR-DRG 3	46.3	43.3	45.3	49.2	47.0
APR-DRG 4	17.8	16.3	14.5	18.0	24.3
Palliative care consultation	12.1	7.1	10.6	14.0	17.4
Number of diagnoses (SD)	13.4 (6.1)	10.8 (5.0)	12.6 (5.7)	14.5 (6.2)	16.2 (6.6)
Palliative procedures	13.5	12.4	13.4	13.3	14.7
LOS, mean (SD), day	7.7 (8.4)	8.2 (8.5)	7.9 (8.7)	7.6 (8.6)	7.1 (7.5)
Total charges, mean $ (SD)	92,052 (13,585)	95,921 (136172)	95,555 (143347)	92,912 (144892)	86,085 (125104)
In-hospital death	7.2	7.9	6.6	6.9	7.5
Hospital characteristics					
Bed size of hospitals					
Small	13.2	12.5	11.7	14.4	16.1
Medium	23.88	22.1	23.1	26.8	26.6
Large	62.92	65.5	65.2	58.8	57.3
Location & teaching hospital					
Urban non-teaching	5.9	7.94	6.45	4.94	4.8
Urban teaching	26.91	37.6	32.84	20.98	15.71
Rural	67.19	54.46	60.71	74.09	79.49
Region					
Northeast	22.73	21.08	23.5	22.28	21.98
Midwest	20.27	22.59	19.85	20.1	19.46
South	35.64	35.05	35.4	36.1	35.98
West	21.36	21.28	21.25	21.52	22.58

Note: data are displayed as %, unless otherwise indicated

*NIS* National Inpatient Sample, *SD* standard deviation, *APR-DRG* all-patient refined diagnosis-related group

Note: For the sake of not making the table too wide, only 4 years of data (every 3 years) are displayed in this table

### Time-trends of hospitalized gastric Cancer patients, palliative care, and palliative procedures

**Fig. 2:**
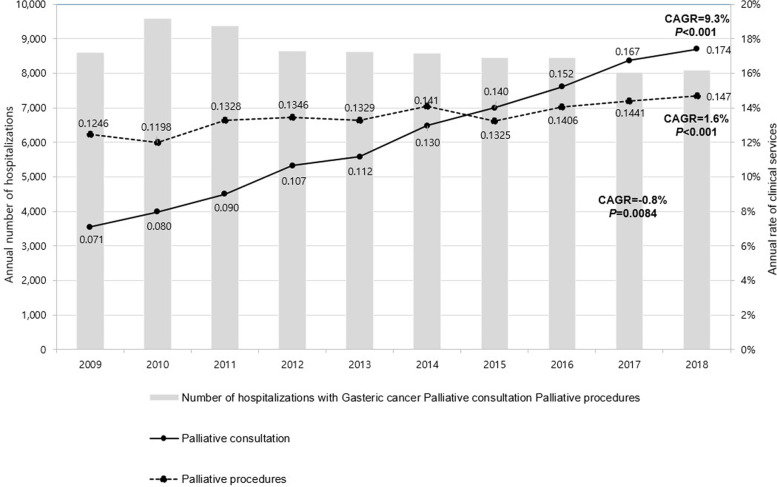
CAGRs of the Annual Incidence of Hospitalization Number, Palliative Care Consultation, and Palliative Procedures for Gastric Cancer Patients. CAGR, Compound Annual Growth Rate. Note: CAGR = (y/x)^[1/(B-A)]-1^, year A is x and year B is y [9, 17]

Figure 2 presents CAGRs of the annual number of hospitalizations with gastric cancer, palliative care consultation, and palliative procedure during 2009–2018. The number of hospitalized patients with gastric cancer gradually decreased (CAGR: -0.8%, *P =* 0.0084). Meanwhile, the utilization of palliative care consultation and palliative procedures significantly increased over the same period. CAGR was 9.3% (*P* < 0.001) for palliative care consultation and 1.6% (*P* < 0.001) for palliative procedures. Compared with palliative procedures, utilization of palliative care consultation appeared to increase more rapidly.

### Factors associated with utilization of palliative care and palliative procedures

Table 2 shows factors associated with palliative care consultation in gastric cancer patients. Palliative care utilization significantly increased over time (odds ratio [OR]: 1.08, 95% confidence interval [CI]: 1.07–1.09). Other positively-associated factors were older age group, female, Hispanic (compared with white), Medicaid user (compared with private insurance), severity of illness, and number of diagnoses. It was remarkable that patients who died in hospital were six times more likely to receive palliative care consultation than discharged-live patients (OR: 6.01, 95% CI: 5.64–6.40). In contrast, Medicare user, small-sized hospital, rural and urban-nonteaching hospital (both compared with urban-teaching hospital) were inversely associated with palliative care consultation (Table 2).

**Table 2:** Multivariate Analysis for Factors Associated with Utilization of Palliative Care Consultation in Gastric Cancer (*n* = 86,430)

Independent variable	Odds ratio	Lower 95% CI	Upper 95% CI	***P***-value
Year	1.08	1.07	1.09	<.0001
Age-group	1.07	1.05	1.09	<.0001
Female	1.26	1.20	1.32	<.0001
Race				
White (reference)	1.00			
Black	1.03	0.97	1.11	0.3455
Hispanic	1.21	1.12	1.30	<.0001
Asian/Pacific Islander	1.11	1.01	1.22	0.0384
Other	1.11	0.98	1.24	0.0895
Primary payer				
Private insurance (reference)	1.00			
Medicare	0.75	0.70	0.80	<.0001
Medicaid	1.19	1.10	1.29	<.0001
Uninsured	1.08	0.94	1.25	0.2619
No charge	0.79	0.52	1.19	0.2552
Other	1.80	1.59	2.04	<.0001
Severity of illness: APR-DRG	1.27	1.22	1.32	<.0001
Number of diagnoses	1.07	1.07	1.08	<.0001
In-hospital death	6.01	5.64	6.40	<.0001
Quartile of median income by zip code	1.02	1.00	1.05	0.0500
Hospital bed size				
Large (reference)	1.00			
Small	0.83	0.77	0.89	<.0001
Medium	0.99	0.93	1.05	0.7938
Hospital location and teaching status				
Urban-teaching (reference)	1.00			
Rural	0.78	0.70	0.87	<.0001
Urban-nonteaching	0.87	0.82	0.92	<.0001
Hospital region				
South (reference)				
Northeast	1.01	0.93	1.10	0.8382
Midwest	1.02	0.95	1.11	0.5550
West	1.05	0.97	1.13	0.2439

*CI* Confidence Interval, *APR-DRG* all patient refined-diagnosis-related group

Factors associated with palliative procedure utilization are summarized in Table 3. Severity of illness, number of diagnoses, median income, and hospital in Northeast area were significantly associated with higher utilization of palliative procedures. Meanwhile, female, black and Hispanic (both compared with white), small and medium sized hospitals (both compared with large hospital), and rural and urban-nonteaching hospitals (both compared with urban-teaching hospital) were significant factors of less utilization of palliative procedures. Interestingly, contrary to results found for palliative care consultation, patients who died in hospitals received less palliative procedures (OR: 0.65, 95% CI: 0.60–0.71) (Table 3).

**Table 3:** Multivariate Analysis for Factors Associated with Utilization of Palliative Procedures in Gastric Cancer (n = 86,430)

Independent variable	Odds ratio	Lower 95% CI	Upper 95% CI	***P***-value
Year	1.00	0.99	1.01	0.9818
Age-group	0.98	0.96	1.00	0.0927
Female	0.84	0.80	0.87	<.0001
Race				
White (reference)	1.00			
Black	0.80	0.75	0.85	<.0001
Hispanic	0.82	0.76	0.87	<.0001
Asian/Pacific Islander	0.92	0.85	1.01	0.0835
Other	0.84	0.76	0.94	0.0015
Primary payer				
Private insurance (reference)	1.00			
Medicare	0.96	0.91	1.02	0.1641
Medicaid	0.96	0.89	1.03	0.2647
Uninsured	0.94	0.82	1.07	0.3622
No charge	0.69	0.47	1.01	0.056
Other	0.93	0.82	1.07	0.329
Severity of illness: APR-DRG	1.58	1.53	1.64	<.0001
Number of diagnoses	1.02	1.01	1.02	<.0001
In-hospital death	0.65	0.60	0.71	<.0001
Quartile of median income by zip code	1.03	1.01	1.05	0.0142
Hospital bed size				
Large (reference)	1.00			
Small	0.73	0.68	0.79	<.0001
Medium	0.80	0.75	0.84	<.0001
Hospital location and teaching status				
Urban-teaching (reference)	1.00			
Rural	0.65	0.59	0.72	<.0001
Urban-nonteaching	0.76	0.72	0.81	<.0001
Hospital region				
South (reference)	1.00			
Northeast	1.30	1.22	1.40	<.0001
Midwest	1.02	0.95	1.09	0.5858
West	1.02	0.95	1.10	0.5179

*CI* Confidence Interval, *APR-DRG* all patient refined-diagnosis-related group

### Factors associated with Total Hospital charges

Table 4 presents results of multivariable regression analysis for total hospital costs. Hospital charges significantly decreased over the years of the study period, shown an average decrease of $3875 USD (inflation adjusted, *P* < 0.0001). Palliative care (−$34,188, *P* < 0.0001) was also significantly associated with lower hospital cost, while palliative procedure was associated with higher cost ($40,123 USD, *P* < 0.0001). Other factors associated with lower hospital costs were older age group, female, black race (compared with white), Medicaid user or uninsured patient (compared with private insurance), small and medium sized hospitals (compared with large hospital), rural and urban-nonteaching hospitals (compared with urban-teaching hospital), and hospitals located in Midwest region (compared with South region). However, Hispanic and Asian-Pacific islander (compared with white), severity of illness, number of diagnoses, died in hospital, and hospitals in Northeast and West region were associated with higher hospital cost (Table 4).

**Table 4:** Multivariate Analysis for Factors Associated with Total Hospital Charges in Gastric Cancer Patients (n = 86,430)

Independent variable	Coefficient, β	Standard error	***p-***value
Year	-3875	216	<.0001
Palliative care consultation	−34,188	1385	<.0001
Palliative procedure	40,123	1251	<.0001
Age-group	− 5136	398	<.0001
Female	− 1648	878	0.0607
Race			
White (reference)			
Black	− 3169	1304	0.0151
Hispanic	6353	1422	<.0001
Asian/Pacific Islander	6023	1885	0.0014
Other	8225	2213	0.0002
Primary payer			
Private insurance (reference)			
Medicare	− 260	1199	0.8282
Medicaid	− 4413	1489	0.0030
Uninsured	− 7770	2649	0.0034
No charge	− 3571	6607	0.5888
Other	− 4259	2730	0.1188
Severity of illness: APR-DRG	37,626	692	<.0001
Number of diagnoses	3422	93	<.0001
Died in hospitals	20,577	17,44	<.0001
Quartile of median income by zip code	393	426	0.3558
Hospital bed size			
Large (reference)			
Small	−20,159	1514	<.0001
Medium	−12,811	1244	<.0001
Hospital location and teaching status			
Urban-teaching (reference)			
Rural	−39,661	2084.02	<.0001
Urban-nonteaching	− 9499.56	1237.25	<.0001
Hospital region			
South (reference)			
Northeast	10,463	1877	<.0001
Midwest	−14,660	1754	<.0001
West	30,606	1840	<.0001

*APR-DRG* all patient refined-diagnosis-related group

## Discussion

Our study presents changing trends of hospitalization status and utilization of palliative care consultation and palliative procedures during the recent 10-year in the US. In addition, it analyzed factors affecting hospital costs from a socioeconomic perspective using a nationwide database. Our study showed that the annual number of hospitalizations showed a slow decrease during 2009–2018, with CAGR of 0.8%. This result was consistent with a previous study showing decreased mortality and hospitalizations of gastric cancer patients in the US [6]. In that study, the authors showed that 23,921 admissions as a primary discharge diagnosis of gastric cancer were detected in 2003 compared with 21,540 in 2014 (*P* < 0.001). In addition, LOS was decreased significantly (10.9 vs. 8.9 days, *P* < 0.001), while mean hospital charges per patient significantly increased from $75,341 in 2003 to $91,385 in 2014 (*P* < 0.0001) [6]. However, the present study did not show such changing trends of palliative care consultation or palliative procedures in patients with gastric cancer. Our study showed that both palliative care consultation and palliative procedures had gradually increased during 2009–2018, although the number of hospitalizations was decreased. To the best of our knowledge, this is the first study to use nationwide data for investigating temporal trends of palliative care consultation and palliative procedures as well as the annual number of hospitalizations among gastric cancer patients in the US. It is expected that patients near end of life (EOL) due to advanced illness usually receive more palliative care consultation with less interventional or life-sustaining procedures such as ventilation, cardiopulmonary resuscitation, blood transfusion, and dialysis [18]. In our study, we limited included procedures to those used only for palliative purpose, such as non-surgical bypass, gastrostomy, enterostomy, dilation, drainage, nutrition, and irrigation, while aggressive life-sustaining procedures were not included. This might have led to parallel increasing trends of utilization of palliative procedures with palliative care consultation. Interestingly, the utilization of palliative care consultation increased more rapidly than that of palliative procedures (CAGR 9.3% vs. CAGR 1.6%), the details of which will need to be explored in the future.

Factors associated with the utilization of palliative care consultation were also analyzed in this study. It was found that older age group, female, Hispanic and Asian-Pacific Islander, and Medicaid users received more palliative care consultation, while patients in small to medium sized hospitals and rural or urban-nonteaching hospitals received significantly less palliative care consultation. These findings are broadly consistent with a previous study investigating determinants of palliative care utilization among patients hospitalized with metastatic gastrointestinal malignancies. In that study, the authors analyzed the NIS database and found that female, Hispanic or African-American, Medicaid (compared with Medicare) user, and large sized and urban-teaching hospitals were associated with inpatient palliative care utilization [19]. It is unclear why gender, racial, and insurance differences were observed in the utilization of palliative care consultation. It could be partially explained by the following findings: 1) males with malignant diseases near EOL might have more risk of emergency department attendance than females [20]; and 2) Medicaid is a commercial insurance with higher self-pay status compared to Medicare users [21]. We also found that the severity of illness and the number of diagnoses were positive factors for palliative care utilization, as expected. Interestingly, in-hospital death was also highly associated with palliative care utilization. Previous studies have shown that introduction of early palliative care has advantages to effectively improve health care utilization by reducing hospital charges and in-hospital mortality in patients with advanced chronic illness and malignant diseases [22–25]. The present study showed similar results to previous studies in terms of hospital costs. However, our result on the association between palliative care and in-hospital mortality was inconsistent with previous studies. Due to the lack of previous data about palliative care consultation in patients with gastric cancer in the US, the cause of discrepancy in such results of in-hospital mortality between ours and previous studies could not be determined. Patients with gastric cancer might be referred to hospitals at a later stage, which might have led to a late introduction of palliative care consultation and an increase of in-hospital mortality because gastric cancer is not a major malignancy in the US and its associated symptoms are vague until it is developed at an advanced stage [26].

Factors associated with the utilization of palliative procedures were also analyzed in this study. It was found that females and black or Hispanic patients were less likely to receive palliative procedures. However, median income by zip code was a positive factor for utilization of palliative procedures. It is generally known that minority patients with EOL are less likely to receive advanced care directives [27], while patients with higher socioeconomic status are more likely to receive it [20], However, our study did not demonstrate significant differences in the utilization of palliative procedures according to the insurance type. Our data of palliative procedures considering hospital characteristics such as hospital bed size and hospital location with teaching status showed very similar pattern to those of palliative care consultation. This suggests that both palliative care consultation and palliative procedures for gastric cancer patients in the US are usually performed in large, urban-teaching hospitals. It is noteworthy that utilization of palliative procedures was negatively associated with in-hospital mortality, in contrast with palliative care consultation. This finding suggests that palliative procedures in gastric cancer patients near EOL may facilitate a transition to improved EOL care outside the hospital setting. This finding deserves further investigation of the actual factors involved.

Our analysis for factors associated with total hospital charges showed that multiple independent variables contributed to the increase or decrease of hospital charges in gastric cancer patients. It was noticeable that hospital charges were significantly decreased over the period of 2009–2018 and that utilization of palliative care was a strong factor for reduced hospital charges. These results suggest that increased utilization of palliative care consultation may reduce unnecessary health care costs. However, we found that utilization of palliative procedure was positively associated with hospital cost, consistent with our previous study showing that systemic or local procedure was associated with higher hospital charges in terminally ill patients [9]. There were gender and racial differences in that females paid less hospital charges while Hispanic and Asian-Pacific Islanders paid significantly more in the present study, consistent with a previous study [19]. Hospital factors were also significantly associated with hospital charges, with large and urban-teaching hospitals showing increased hospital charges, which was expected.

### Study limitations

This study has several limitations. First, we included palliative care consultation only based on palliative care (ICD-9 code: V66.7, ICD-10 code: Z51.5) and advance care planning (ICD-9 code: V69.89, ICD-10 code: Z71.89) codes, which might have led to a low sensitivity due to missing data. For example, a recent validation study of the V66.7 code for palliative care consultation has shown a low sensitivity (66.3%) among metastatic cancer patients, although it showed an optimal specificity (99.1%) [10]. Second, palliative care can be supplied in outpatient or home-based settings as well as inpatient settings. However, out study cohort did not include home-based palliative care population. Thus, results of this study cannot be applied to outpatient or home-based palliative care settings [12]. Third, we captured gastric cancer cases only based on the ICD-9 and ICD-10 codes. Thus, we could not differentiate stages of gastric cancer patients. In addition, since NIS does not have a variable to distinguish a recent diagnosis from all other diagnoses of gastric caner, we might have included early staged gastric cancer cases not relevant to our study. Finally, as we relied on ICD codes for detecting palliative procedures, selected procedures might be actually performed for life-sustaining purpose rather than palliative purpose.

## Conclusions

Despite above-mentioned limitations, our study revealed that palliative care consultation for gastric cancer patients increased during 2009–2018 in the US, although the number of hospitalized patients was decreased. Associated with the increase in palliative care consultation was a reduction of hospital cost. Further study may determine if it is possible that use of early palliative care referral near EOL may further improve the effective distribution of medical resources and reduce hospital costs.

## Supplementary Information

**Additional file 1: Supplementary Table 1**. ICD-9-CM and ICD-10-PCS Codes used for Palliative Procedure and Palliative Care.

## Data Availability

The datasets generated and/or analysed during the current study are available in the NIS database, https://www.hcup-us.ahrq.gov. Also, are available from the corresponding author on reasonable request.

## Abbreviations

EGC: Early gastric cancer
EOL: End of life
NIS: National Inpatient Sample
HCUP: Healthcare charge and Utilization Project
AHRQ: Healthcare Research and Quality
IRB: Institutional Review Board
UNLV: University of Nevada Las Vegas
ICD-CM: International Classification of Diseases Clinical Modification
APR-DRG: All Patient Refined Diagnosis-Related Group
LOS: Length of stay
CAGR: Compound annual growth rate
OR: Odds ratios
CI: Confidence intervals
