# Supplementary material for: Ten-year trends of utilizing palliative care and palliative procedures in patients with gastric Cancer in the United States from 2009 to 2018 - a nationwide database study
Source: BMC Health Serv Res. 2022 Jan 4;22:20. doi: 10.1186/s12913-021-07404-1 (PMC8725552; doi:10.1186/s12913-021-07404-1)
Supplement: Supplementary file 1 — Additional file 1: Supplementary Table 1. ICD-9-CM and ICD-10-PCS Codes used for Palliative Procedure and Palliative Care. [file 12913_2021_7404_MOESM1_ESM.docx]

**Supplementary Table 1**. ICD-9-CM and ICD-10-PCS Codes used for Palliative Procedure and Palliative Care

| **Category** | **Subcategory** | **ICD-9 code** | **ICD-10 code** |
| --- | --- | --- | --- |
| Palliative procedure | Bypass, gastrostomy and enterostomy | 43.11, 44.32, 46.32, 97.01, 97.02, 97.03, 97.05, 97.51, 97.52, 97.55, 97.59 | 0D16874, 0D16879, 0D1687A, 0D1687B, 0D1687L, 0D168J4, 0D168J9, 0D168JA, 0D168JB, 0D168JL, 0D168K4, 0D168K9, 0D168KA, 0D168KB, 0D168KL, 0D168Z4, 0D168Z9, 0D168ZA, 0D168ZB, 0D168ZL, 0DH63DZ, 0DH63UZ, 0DH63YZ, 0DH64DZ, 0DH64UZ, 0DH64YZ, 0DH67DZ, 0DH67UZ, 0DH67YZ, 0DH68DZ, 0DH68UZ, 0DH68YZ. 0DH83UZ, 0DH84UZ, 0DH87UZ, 0DH88UZ, 0DH93UZ, 0DH94UZ, 0DH97UZ, 0DH98UZ, 0DHA3UZ, 0DHA4UZ, 0HDA7UZ, 0DHA8UZ |
|  | Dilation | 42.92, 44.22 | 0D743DZ, 0D743ZZ, 0D744DZ, 0D744ZZ, 0D747DZ, 0D747ZZ, 0D748DZ, 0D748ZZ, 0D763DZ, 0D763ZZ, 0D764DZ, 0D764ZZ, 0D767DZ, 0D767ZZ, 0D768DZ, 0D768ZZ, 0D773DZ, 0D773ZZ, 0D774DZ, 0D774ZZ, 0D777DZ, 0D777ZZ, 0D778DZ, 0D778ZZ |
|  | Drainage | 51.43, 54.91, 97.82 | 0D9430Z, 0D943ZX, 0D943ZZ, 0D9440Z, 0D944ZX, 0D944ZZ, 0D9470Z, 0D947ZX, 0D947ZZ, 0D9480Z, 0D948ZX, 0D948ZZ, 0D9630Z, 0D963ZX, 0D963ZZ, 0D9640Z, 0D964ZX, 0D964ZZ, 0D9670Z, 0D967ZX, 0D967ZZ, 0D9680Z, 0D968ZX, 0D968ZZ, 0D9730Z, 0D973ZX, 0D973ZZ, 0D9740Z, 0D974ZX, 0D974ZZ, 0D9770Z, 0D977ZX, 0D977ZZ, 0D9780Z, 0D978ZX, 0D978ZZ, 0D9870Z, 0D9880Z, 0D20X0Z, 0D20XYZ, 0F9030Z, 0F903ZZ, 0F9040Z, 0F904ZZ, 0FHB3DZ, 0FHB4DZ, 0FHB7DZ, 0FHB8DZ, 0W2GX0Z, 0W2GXYZ, 0WHG3YZ, 0WHG4YZ, 0WPG30Z, 0WPG3YZ, 0WPG40Z, 0WPG4YZ, 0WPGX0Z |
|  | Nutrition | 96.07, 96.08, 96.6 | 3E0G36Z, 3E0G4GC, 3E0G76Z, 3E0G86Z, 3E0G7GC, 3E0G8GC, 0D20XUZ |
|  | Irrigation | 96.31, 96.32, 96.33, 96.34, 96.35, 96.36 | 3C1ZX8Z, 3E1G78Z, 3EG88Z |
| Palliative care | Palliative care | V66.7 | Z51.5 |
|  | Advance care planning | V69.89 | Z71.89 |

ICD-9-CM, International Classification of Diseases, 9^th^ revision, Clinical Modification; ICD-10-PCS, International Classification of Diseases, 10^th^ revision, Procedure Coding System
